# Structural and Mutational Analysis of Functional Differentiation between Synaptotagmins-1 and -7

**DOI:** 10.1371/journal.pone.0012544

**Published:** 2010-09-02

**Authors:** Mingshan Xue, Timothy K. Craig, Ok-Ho Shin, Liyi Li, Chad A. Brautigam, Diana R. Tomchick, Thomas C. Südhof, Christian Rosenmund, Josep Rizo

**Affiliations:** 1 Department of Neuroscience, Baylor College of Medicine, Houston, Texas, United States of America; 2 Department of Biochemistry, University of Texas Southwestern Medical Center, Dallas, Texas, United States of America; 3 Department of Pharmacology, University of Texas Southwestern Medical Center, Dallas, Texas, United States of America; 4 Department of Neuroscience and Cell Biology, University of Texas Medical Branch, Galveston, Texas, United States of America; 5 Department of Molecular Genetics, University of Texas Southwestern Medical Center, Dallas, Texas, United States of America; 6 Department of Molecular and Cellular Physiology, Stanford University, Palo Alto, California, United States of America; 7 Howard Hughes Medical Institute, Stanford University, Palo Alto, California, United States of America; 8 Department of Molecular and Human Genetics, Baylor College of Medicine, Houston, Texas, United States of America; 9 Neuroscience Research Center, Charite-Universitätsmedizin Berlin, Berlin, Germany; 10 Cluster of Excellence NeuroCure, Charite-Universitätsmedizin Berlin, Berlin, Germany; University of Queensland, Australia

## Abstract

Synaptotagmins are known to mediate diverse forms of Ca^2+^-triggered exocytosis through their C_2_ domains, but the principles underlying functional differentiation among them are unclear. Synaptotagmin-1 functions as a Ca^2+^ sensor in neurotransmitter release at central nervous system synapses, but synaptotagmin-7 does not, and yet both isoforms act as Ca^2+^ sensors in chromaffin cells. To shed light into this apparent paradox, we have performed rescue experiments in neurons from synaptotagmin-1 knockout mice using a chimera that contains the synaptotagmin-1 sequence with its C_2_B domain replaced by the synaptotagmin-7 C_2_B domain (Syt1/7). Rescue was not achieved either with the WT Syt1/7 chimera or with nine mutants where residues that are distinct in synaptotagmin-7 were restored to those present in synaptotagmin-1. To investigate whether these results arise because of unique conformational features of the synaptotagmin-7 C_2_B domain, we determined its crystal structure at 1.44 Å resolution. The synaptotagmin-7 C_2_B domain structure is very similar to that of the synaptotagmin-1 C_2_B domain and contains three Ca^2+^-binding sites. Two of the Ca^2+^-binding sites of the synaptotagmin-7 C_2_B domain are also present in the synaptotagmin-1 C_2_B domain and have analogous ligands to those determined for the latter by NMR spectroscopy, suggesting that a discrepancy observed in a crystal structure of the synaptotagmin-1 C_2_B domain arose from crystal contacts. Overall, our results suggest that functional differentiation in synaptotagmins arises in part from subtle sequence changes that yield dramatic functional differences.

## Introduction

The release of hormones and neurotransmitters by Ca^2+^-triggered exocytosis mediates a wide variety of biological processes, including for instance interneuronal communication and the control of diverse cardiovascular functions. Exocytosis at different types of secretory cells is governed by similar protein machineries that are fine tuned for specific regulatory requirements [1]–[3]. Members from the synaptotagmin family form a hierarchy of Ca^2+^ sensors and are believed to underlie at least in part the distinct Ca^2+^ dependencies of different forms of regulated secretion [4]. Thus, synaptotagmin-1 is the Ca^2+^ sensor that triggers fast neurotransmitter release at the hippocampus [5], [6], while fast release is triggered in other brain regions by this same isoform or by the closely related synaptotagmins-2 or -9 [7], [8]. In contrast, insulin secretion in pancreatic β cells depends on synaptotagmin-7 [9], and acrosomal exocytosis in sperm appears to involve synaptotagmin-6 [10]. In PC12 cells, Ca^2+^-dependent secretion is mediated by synaptotagmins-1 and -9 [11], whereas chromaffin cells use synaptotagmins-1 and –7 as Ca^2+^ sensors [12].

Functional differentiation among synaptotagmins is expected to arise primarily from differences in the properties of the two C_2_ domains that form most of their cytoplasmic regions (referred to as C_2_A and C_2_B domains). In this context, the C_2_ domains of synaptotagmin-1 have been the subjects of particularly extensive study. Both domains form characteristic β-sandwich structures that bind three or two Ca^2+^ ions (C_2_A or C_2_B, respectively) through loops located at the top of the β-sandwich, and do not undergo substantial conformational changes upon Ca^2+^ binding [13]–[17]. While the structures of both domains are similar, the synaptotagmin-1 C_2_B domain contains two α-helices that are not present in the C_2_A domain; one of these helices (HA) is generally found in C_2_ domains of tandem C_2_ domain-proteins [18], while the other helix (HB) is predicted to be present in only synaptotagmins-1, -2 and -8 [17]. Both C_2_ domains bind to phospholipids in a Ca^2+^-dependent manner through the Ca^2+^-binding loops at the top face [19], [20], and changes in the apparent Ca^2+^ affinity of this activity lead to parallel changes in the Ca^2+^ dependence of neurotransmitter release [5], [6]. However, phospholipid binding alone cannot explain the observation that Ca^2+^ binding to the C_2_B domain is much more critical for release than Ca^2+^ binding to the C_2_A domain [21]–[24]. A potential explanation for this finding was provided by the observation that the C_2_B domain (but not the C_2_A domain) can bind to membranes through the bottom face, which allows simultaneous binding to two closely apposed membranes and suggests that synaptotagmin-1 may trigger neurotransmitter release by bringing the synaptic vesicle and plasma membranes together in a Ca^2+^-dependent manner [25], [26]. This proposal has been supported by the finding that mutation of two arginines at the bottom face of the C_2_B domain practically abolishes synaptotagmin-1 function [27]. Moreover, synaptotagmin-1 binds to the SNARE complex that forms part of the core of the membrane fusion machinery that mediates release [28], [29], and this interaction is primarily mediated by the C_2_B domain [30]–[32].

Based on the sequence similarities between the synaptotagmin-1 C_2_ domains and those from other synaptotagmin isoforms and other C_2_ domain-containing proteins involved in membrane traffic, it seems likely that many of these C_2_ domains share at least some of the properties found in synaptotagmin-1 – see [33]-[35] for review. However, the C_2_ domains of some of these proteins lack a full complement of predicted Ca^2+^ ligands and, as a consequence, do not bind Ca^2+^ or phospholipids; instead they act as protein-protein interaction domains [e.g. the Munc13-1 C_2_A domain [36], [37]]. Moreover, even some C_2_ domains with high sequence similarity with the synaptotagmin-1 C_2_ domains can have dramatically different properties. For instance, the piccolo/aczonin C_2_A domain exhibits a dramatic Ca^2+^-dependent conformational change [38], and the synaptotagmin-4 C_2_B domain does not bind Ca^2+^, despite having all the predicted Ca^2+^-binding ligands, because of conformational differences with respect to the synaptotagmin-1 C_2_B domain in the Ca^2+^-binding region [39]. Hence, it is difficult to reliably predict the properties of C_2_ domains based on sequence analyses alone, and the basis for functional differences among synaptotagmins is poorly understood. The relation between the functions of synaptotagmins-1 and-7 is particularly fascinating, as data obtained in chromaffin cells [12] suggest that these two isoforms play at least partially redundant roles, and yet synaptotagmin-7 does not function as a Ca^2+^ sensor for synaptic vesicle exocytosis despite being enriched in synapses [40]. These findings do not arise from difference in the SNAREs, since the same SNARE isoforms mediate exocytosis in both systems - see for instance [41], [42]. Note also that synaptotagmin-7 cannot rescue fast neurotransmitter release in neurons of *synaptotagmin-1* knockout (KO) mice, unlike synaptotagmins-2 and -9 [8].

In this paper, we describe attempts to shed light onto this apparent paradox through a combination of functional and structural studies. We find that a chimeric protein containing the synaptotagmin-1 sequence, but with the C_2_B domain replaced by the synaptotagmin-7 C_2_B domain, is also unable to rescue fast neurotransmitter release in synaptotagmin-1 KO neurons. Even extensive mutagenesis of this chimera to make the C_2_B domain more similar to that of synaptotagmin-1 did not lead to rescue of fast release. Furthermore, deletion of the HB helix in synaptotagmin-1 led to a gain of function, showing that the absence of this helix in synaptotagmin-7 does not underlie its inability to rescue fast release. We describe the crystal structure of the Ca^2+^-bound synaptotagmin-7 C_2_B domain at 1.44 Å resolution, which is very similar to that of the synaptotagmin-1 C_2_B domain. The synaptotagmin-7 C_2_B domain structure also shows that this domain binds three Ca^2+^ ions and that coordination of two of the Ca^2+^ ions is analogous to that found previously in solution by NMR spectroscopy for the synaptotagmin-1 C_2_B domain [17], suggesting that differences in Ca^2+^ coordination observed in a crystal structure of the latter [43] are due to distortions caused by crystal contacts. Overall, our data further support the importance of the C_2_B domain for synaptotagmin-1 function. Although we cannot completely rule out the possibility that the lack of rescue of fast release by the Syt1/7 chimeras might be due to problems with proper targeting to synaptic vesicles and/or protein folding, our data suggest that the functional differences between the synaptotagmin-1 and –7 C_2_B domains do not arise from conformational differences but rather from subtle alterations in biochemical properties caused by multiple residue substitutions. These results also illustrate how it is much more difficult to confer a specific functional activity of a protein to a closely related isoform than to disrupt this activity.

## Results

### Replacing the synaptotagmin-1 C_2_B domain with the synaptotagmin-7 C_2_B domain abolishes rescue of neurotransmitter release

**Figure 1:**
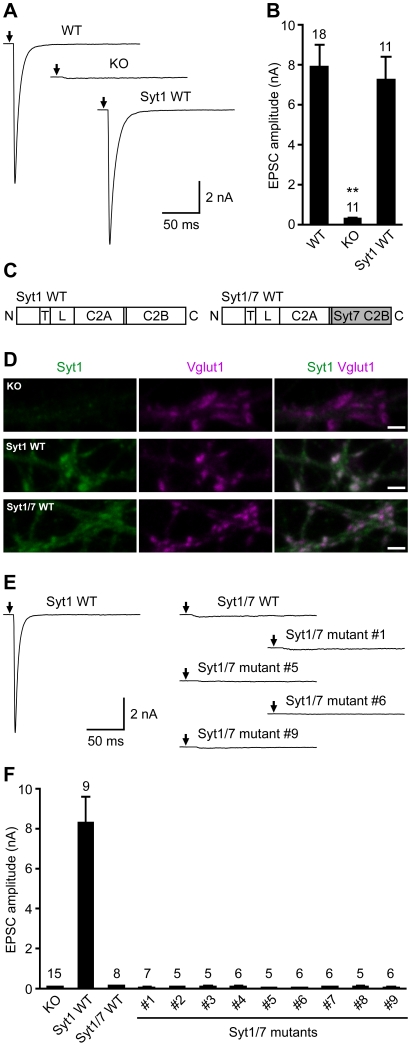
The synapotagmin-7 C2B domain cannot replace the synapotagmin-1 C2B domain to trigger Ca^2+^-evoked fast neurotransmitter release in hippocampal neurons. (A,B) Ca^2+^-triggered fast neurotransmitter release in wild-type neurons (WT), *synapotagmin-1* knockout neurons (KO), and KO neurons rescued by WT synapotagmin-1 (Syt1 WT). (A) Exemplary traces showing the evoked EPSCs. The arrows represent stimulations; artifacts and action potentials are blanked. (B) Bar graphs showing the summary data of EPSC amplitudes. Data are expressed as mean ± SEM. The numbers of neurons analyzed are shown above the bars. **, *P*<0.001 compared to WT or Syt1 WT, one-way analysis of variance. (C) Schematic diagrams illustrating the domain structures of WT synapotagmin-1 (Syt1 WT) and a chimeric synapotagmin-1/7 (Syt1/7 WT) in which the synapotagmin-1 C2B domain (residues 266–421) is replaced by the synapotagmin-7 C2B domain (residues 260–403). N, N terminus; T, transmembrane domain; L, linker region; C, C terminus. (D) Exemplary confocal images showing the presynaptic localization of Syt1 WT and Syt1/7 WT in *synapotagmin-1* KO neurons. Neurons were immunostained with antibodies against the N-terminal portion of the synaptotagmin-1 C2A domain (green images), Vglut1 (magenta images), and EGFP (not shown). The merged images show the presence of synaptotagmin-1 WT and Syt1/7 WT in the presynaptic termini identified by Vglut1. Scale bars: 2 µm. (E,F) Ca^2+^-triggered fast neurotransmitter release in *synapotagmin-1* KO neurons rescued by Syt1 WT or chimeric Syt1/7 variants. The details of the Syt1/7 chimeric constructs are shown in Figure 2C. (E) Exemplary traces showing the evoked EPSCs. The arrows represent stimulations; artifacts and action potentials are blanked. (F) Bar graphs showing the summary data of EPSC amplitudes. Data are expressed as mean ± SEM. The numbers of neurons analyzed are shown above the bars. None of the chimeric Syt1/7 variants is able to restore the diminished EPSC amplitude of *synapotagmin-1* KO neurons (*P*<0.001 compared to Syt1 WT, one-way analysis of variance).

To investigate the basis for functional differentiation between synaptotagmins-1 and -7, we used a rescue approach with autaptic hippocampal glutamatergic neurons from synaptotagmin-1 KO mice. The approach is illustrated in Figures 1A,B, which show that excitatory postsynaptic currents (EPSCs) evoked by action potentials are drastically reduced in synaptotagmin-1 KO neurons (KO) compared to neurons from wild-type (WT) mice, and that normal EPSCs can be obtained upon rescue of the synaptotagmin-1 KO neurons with lentiviral expression of WT synaptotagmin-1 (Syt1 WT). Since synaptotagmin-7 cannot rescue the function of synaptotagmin-1 in neurons [8], and Ca^2+^ binding to the C_2_B domain is more critical than Ca^2+^ binding to the C_2_A domain for synaptotagmin-1 function [21]–[24], we investigated whether replacement of the synaptotagmin-1 C_2_B domain with the synaptotagmin-7 C_2_B domain allows or abolishes rescue. For this purpose, we constructed a lentiviral vector expressing a chimeric protein that contains most of the synaptotagmin-1 sequence, but with the C_2_B domain replaced by the synaptotagmin-7 C_2_B domain (Syt1/7 WT; see domain diagram in Figure 1C). Immunocytochemistry showed that this chimeric protein exhibits a normal synaptic localization, similar to that of WT synaptotagmin-1 (Fig. 1D). However, the Syt1/7 chimeric protein was unable to rescue the impairment of fast neurotransmitter release observed in synaptotagmin-1 KO neurons (Figures 1E,F).

**Figure 2:**
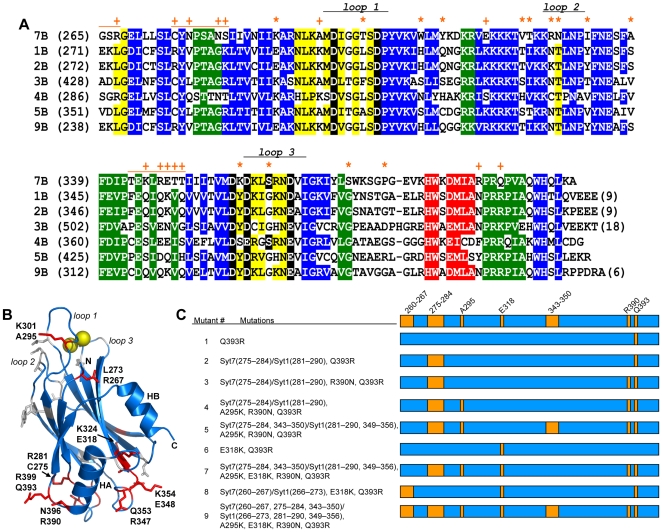
Sequence differences between the C_2_B domains of synaptotagmins-1 and -7. (A) Sequence alignment of the C_2_B domains from selected rat synaptotagmin isoforms illustrating the similarities and differences among them. The first residue number of each sequence in each line is indicated in parenthesis. Conserved residues are color-coded: blue, β-strands; red, helix HA; yellow, top loops; green, bottom loops; black, Ca^2+^ ligands. The positions of the three Ca^2+^-binding loops (loops 1–3) are indicated. Residues that are identical in the C_2_B domains of synaptotagmins-1, -2 and -9 (the three isoforms that can support fast neurotransmitter release in cortical synapses [8]), but are different in the C_2_B domain of synaptotagmin-7, are indicated by a + sign if they were mutated in the Syt1/7 chimeras (see panel C), or by a * otherwise. The orange lines above the synaptotagmin-7 C_2_B domain sequence indicate the positions of fragments in the linker or loop sequences that were replaced with those of synaptotagmin-1 in the Syt1/7 chimeras (see panel C). (B) Ribbon diagram of the NMR structure of the synaptotagmin-1 C_2_B domain [17] illustrating the positions of selected residues mutated in the Syt1/7 chimers (shown as red stick models). The corresponding residue names (in single letter amino acid code) and numbers in synaptotagmin-1 are indicated above, and those in synaptotagmin-7 are indicated below. The bound Ca^2+^ ions are shown as yellow spheres. The positions of the Ca^2+^-binding loops and the two helices (HA and HB) are indicated. (C) Summary of chimeric Syt1/7 mutants used in this study. As illustrated in Figure 1C, Syt1/7 WT is composed of residues 1–265 of synaptotagmin-1 and the WT C2B domain (residues 260–403) of synaptotagmin-7. Each of the Syt1/7 mutants is composed of the same residues of synaptotagmin-1 (residues 1–265) and synaptotagmin-7 (residues 260–403) (represented in blue in the diagrams), but with the indicated mutations (illustrated by the orange boxes in the diagrams). The residue numbers of mutations are based on the respective WT synaptotagmins-1 and -7. In addition to point mutations, several stretches of residues in the synaptotagmin-7 C2B domain were changed to the corresponding residues in the synaptotagmin-1 C2B domain. These changes are listed as Syt7(residues)/Syt1(residues). For example, in Syt1/7 mutant #5, the synaptotagmin-7 residues 275–284 and 343–350 were replaced by the synaptotagmin-1 residues 281–290 and 349–356, respectively.

These results confirmed the importance of the C_2_B domain for synaptotagmin-1 function and led us to use the Syt1/7 chimera as a benchmark to investigate which residues of the synaptotagmin-1 C_2_B domain are fundamental for its role in synchronous neurotransmitter release, yielding crucial functional differences with the synaptotagmin-7 C_2_B domain. Figure 2A shows a sequence alignment of the C_2_B domains from various synaptotagmin isoforms that illustrates the high sequence similarities among them, and Figure 2B shows the structure of the synaptotagmin-1 C_2_B domain [17] highlighting some of the residues that are different in synaptotagmin-7. Comparing the synaptotagmins-1 and -7 C_2_B domains, 48% of their residues are identical, and many of the differences between them represent conservative substitutions. Note that some of the positions exhibiting differences are also distinct in synaptotagmins-2 and/or -9, which can rescue release in synaptotagmin-1 KO neurons [8]. Hence, these differences are unlikely to underlie the lack of rescue by synaptotagmin-7 or the Syt1/7 chimera. It also seems unlikely that such lack of rescue arises from sequence differences in the Ca^2+^-binding loops at the top of the C_2_B domain (loops 1–3, see Figure 2B), since their sequences are highly similar in synaptotagmins-1, -2, -7 and -9 (Figure 2A), and synaptotagmin-7 is highly efficient in Ca^2+^-dependent phospholipid binding, which constitutes the primary activity of these loops [44], [45]. Based on these arguments, and considering that ionic interactions are important for the biochemical properties of the synaptotagmin C_2_ domains [20], [29], we attempted to recover synaptotagmin-1-like function in the Syt1/7 chimera by focusing on differences between the synaptotagmin-1 and -7 C_2_B domains involving charged residues or loops that are substantially different in synaptotagmin-7, compared to synaptotagmins-1, -2 and -9.

A summary of the mutants of the Syt1/7 chimera that we generated is shown in Figure 2C, and Figure 2B shows the locations of some of the mutated residues on the structure of the synaptotagmin-1 C_2_B domain. In all these mutants, residues from the synaptotagmin-7 C_2_B domain sequence present in the chimera were replaced with the corresponding residues of synaptotagmin-1 to make the chimera more ‘synaptotagmin-1 like’. For instance, in mutant #1, Q393 at the bottom of the synaptotagmin-7 C_2_B domain was replaced with an arginine, since the corresponding residue in synaptotagmin-1 is arginine (R399) and an R399Q mutation strongly impairs synaptotagmin-1 function [27]. Other point mutations involving charge differences included: R390, which is close to Q393 at the bottom of the C_2_B domain and is asparagine (N396) in synaptotagmin-1; A295, which is at the top of the C_2_B domain and is lysine (K301) in synaptotagmin-1; and E318, which is in the middle of a characteristic polybasic region at the side of the C_2_B domain and is lysine (K324) in synaptotagmin-1. We also replaced two loops at the bottom of the C_2_B domain (residues 275–284 and 343–350 of synaptotagmin-7, which correspond to residues 281–290 and 349–356, respectively, of synaptotagmin-1), as well as the linker region between the C_2_A and C_2_B domains (residues 260–267 of synaptotagmin-7, corresponding to residues 266–273 of synaptotagmin-1) (Figure 2C). Immunocytochemistry revealed that all mutants were correctly targeted to synapses (data not shown), as observed for the WT Syt1/7 chimera (Fig. 1D). However, none of these mutations, whether performed individually or in combination, was able to endow the Syt1/7 chimera with the ability to rescue fast Ca^2+^-triggered neurotransmitter release in synaptotagmin-1 KO neurons (Figures 1E,F). It is indeed remarkable that such ability was not recovered at least in part even in mutant #9, since 30 residues were restored back to those present in synaptotagmin-1 in this mutant, and these residues involve the major obvious differences between the sequences of the synaptotagmins-7 and -1 C_2_B domains.

### Inhibitory role of the HB helix in synaptotagmin-1 function

**Figure 3:**
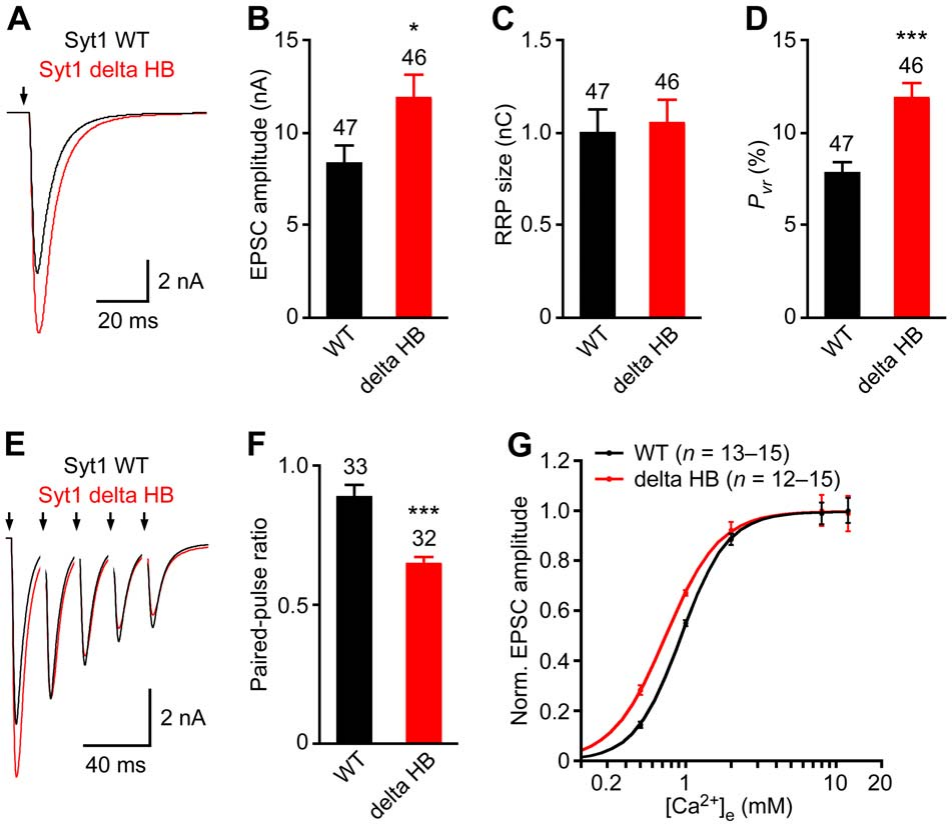
Helix HB plays an inhibitory role in fast Ca^2+^-triggered neurotransmitter release. Hippocampal *synaptotagmin-1* KO neurons were rescued with WT or delta HB mutant synaptotagmin-1. (A,B) Average traces (A) and summary data (B) of basal EPSCs evoked by action potentials at 0.2 Hz. The arrows represent stimulations; artifacts and action potentials are blanked. (C) Readily releasable vesicle pool (RRP) size was estimated by measuring the charge of the transient synaptic current induced by hypertonic sucrose solution. (D) Vesicular release probability (*P_vr_*) was calculated by the ratio of evoked EPSC charge and RRP size. (E) Average traces of 5 consecutive EPSCs evoked at 50 Hz. (F) Paired-pulse ratio was calculated by the ratio of the second EPSC amplitude and the first EPSC amplitude, which were evoked at 50 Hz. (G) Apparent Ca^2+^-sensitivity of evoked release. EPSC amplitudes were normalized by the maximal response and plotted against external Ca^2+^ concentrations ([Ca^2+^]_e_). Data were fitted with standard Hill equation to obtain dissociation constant (*K_d_*). WT, *K_d_* = 0.93±0.01 mM, delta HB, *K_d_* = 0.74±0.01 mM (*P*<0.05). All data are expressed as mean ± SEM. The numbers of neurons analyzed are shown above the bars. *, *P*<0.05; ***, *P*<0.0001 compared WT Syt1 (Student's *t*-test for panels B, C, D, and F; two-way analysis of variance for panel G).

The structure of the rabphilin C_2_B domain showed that a major structural difference between the C_2_A and C_2_B domains of tandem C_2_ domain proteins is the presence of helix HA at the bottom of the β-sandwich [18]. It was later shown that the synaptotagmin-1 C_2_B domain contains an additional helix at the C-terminus (helix HB) that is predicted to be present only in synaptotagmins-1, -2 and -8 [17]. Indeed, it has been proposed that this helix might be critical for synaptotagmin-1 function [46], and the absence of helix HB in the synaptotagmin-7 C_2_B domain could explain its inability to rescue release in synaptotagmin-1 KO neurons. To test this hypothesis, we prepared a lentiviral vector expressing a synaptotagmin-1 fragment lacking the HB helix (Syt1 delta HB, containing residues 1–409) and performed additional rescue experiments in synaptotagmin-1 KO neurons. Western blotting and immunocytochemistry showed that Syt1 delta HB is expressed at similar levels as WT synaptotagmin-1 and properly targets to presynaptic terminals in synaptotagmin-1 KO neurons (Figure S1). Importantly, deletion of the HB helix leads to a significant increase in the EPSC amplitude (Figures 3A,B). Measurement of the readily-releasable pool (RRP) of vesicles using hypertonic sucrose solution showed that vesicle priming is not affected by deletion of the HB helix (Figure 3C). Hence, the deletion leads to an increase in the vesicular release probability, measured as the ratio between EPSC charge and the RRP size (Figure 3D). To further test this conclusion, we compared the responses obtained upon repetitive stimulation, and found that the delta HB mutation leads to a significant decrease in the paired-pulse ratio compared to WT synaptotagmin-1 (Figures 3E,F), as expected for an increase in the release probability. Finally, we also measured the dependence of the EPSC amplitude on the extracellular Ca^2+^ concentration and found that the HB delta mutant exhibits a significant increase in the apparent Ca^2+^ sensitivity of release (Figure 3G). Hence, these results show that the HB helix plays an inhibitory role in release and that its deletion leads to a gain of function, indicating that the absence of this helix in synaptotagmin-7 cannot explain its inability to rescue neurotransmitter release in *synaptotagmin-1* KO neurons.

### Crystal structure of the synaptotagmin-7 C_2_B domain

The crystal structure of the synaptotagmin-4 C_2_B domain showed substantial conformational differences with respect to the synaptotagmin-1 C_2_B domain; these differences were unexpected, given the high similarities between their sequences, and explained the lack of Ca^2+^ binding to the synaptotagmin-4 C_2_B domain [39]. Hence, we hypothesized that the inactivity of the Syt1/7 chimera and its diverse mutants in the rescue experiments with synaptotagmin-1 KO neurons (Figure 1) might arise from conformational differences between the C_2_B domains of synaptotagmins-1 and -7. To test this hypothesis, we performed crystallization screens with the isolated synaptotagmin-7 C_2_B domain and were able to obtain crystals in the presence of Ca^2+^ that diffracted to 1.44 Å. The structure of the synaptotagmin-7 C_2_B domain was determined by molecular replacement using a crystal structure of the synaptotagmin-1 C_2_B domain [43] (PDB accession code 1TJM) to generate a search model. The data collection and refinement statistics are described in Table 1.

**Table 1:** Refinement Statistics for the crystal structure of the synaptotagmin-7 C_2_B domain.

**Data Collection**	
Space Group	P2_1_2_1_2_1_
Cell Dimensions a, b, c (Å)	35.60, 44.89, 87.38
Wavelength (Å)	1.0148
Resolution Range (Å)	50−1.44 (1.46−1.44)
Rsym (%)	3.9 (39.7)
I/σ	31.1 (2.6)
Completeness (%)	98.0 (91.0)
Redundancy	4.7 (4.0)
No. Reflections	25,581 (1,158)
**Refinement**	
Resolution range (Å)	26.6−1.44 (1.50−1.44)
Rwork (%)	16.0 (19.3)
Rfree (%)	18.1 (24.0)
**No. Atoms**	
Non-H Protein/Solvent/Ca^2+^	1,243/183/4
Average B-value (Å2)	14.3/28.0/12.0
**RMS deviations**	
Bond Lengths (Å)	0.021
Bond Angles (°)	1.34

**Figure 4:**
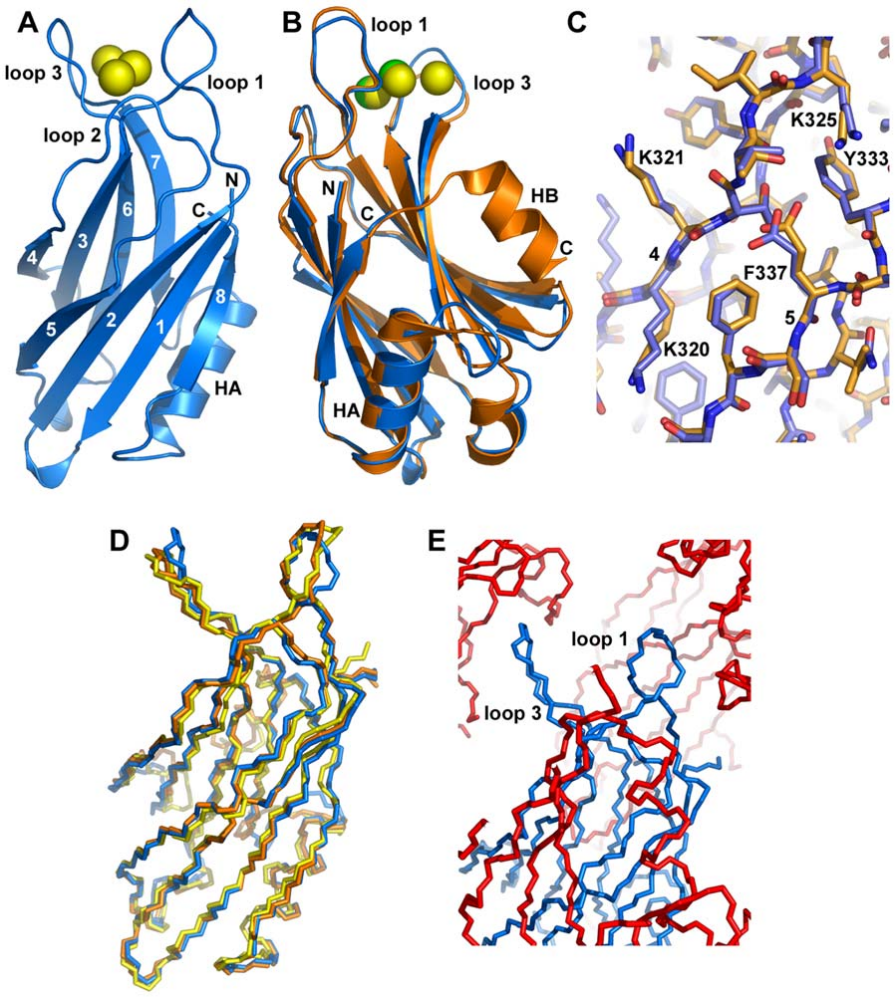
Crystal structure of the synaptotagmin-7 C_2_B domain. (A) Ribbon diagram of the crystal structure of the synaptotagmin-7 C_2_B domain with the β-strands numbered from 1 to 8. The positions of helix HA and the Ca^2+^-binding loops are indicated. Bound Ca^2+^ ions are shown as yellow spheres. The N- and C-termini are labeled. (B) Superposition of ribbon diagrams of the crystal structures of synaptotagmin-7 C_2_B domain (blue) and the synaptotagmin-1 C_2_B domain [43] (PDB accession code 1UOW) (orange). The positions of α-helices HA and HB are indicated. (C) Stick models of the same superposition shown in panel (B) focusing on a region containing β-strands 4 and 5 (labeled with the corresponding numbers). Carbon atoms are colored in blue or orange for the C_2_B domains of synaptotagmin-7 and -1, respectively. Oxygen atoms are red and nitrogen atoms are blue in both structures. Selected side chains from the synaptotagmin-7 C_2_B domain are labeled. (D) Superposition of backbone stick models of the crystal structure of the synaptotagmin-7 C_2_B domain (blue), and the structures of the synaptotagmin-1 C_2_B domain determined by X-ray crystallography [43] (orange) or NMR spectroscopy [17] (PDB accession code 1K5W) (yellow). (E) Backbone stick models illustrating the extensive crystal contacts involving the Ca^2+^-binding loops of the synaptotagmin-7 C_2_B domain. The central molecule is colored in blue, with the positions of loops 1 and 3 indicated. Symmetry-related molecules within the crystal that contact the Ca^2+^-binding loops of this molecule are shown in red. The observed contacts are a result of crystal packing.

Figure 4A shows a ribbon diagram of the structure of the synaptotagmin-7 C_2_B domain, which consists of the β-sandwich characteristic of C_2_ domains and, as expected, includes the HA helix but not the HB helix observed in the synaptotagmin-1 C_2_B domain (see Figure 2B). Apart from this expected difference, the structure of the synaptotagmin-7 C_2_B domain is very similar to the crystal structure of the synaptotagmin-1 C_2_B domain [43] (Figure 4B). This high similarity includes the side chains, even for surface-exposed side chains that would be expected to have some degree of flexibility (e.g. K320 and K321 of synaptotagmin-7, corresponding to K327 and K328 of synaptotagmin-1, respectively; see Figure 4C). The r.m.s. deviation between 713 common atoms of the synaptotagmins-7 and -1 C_2_B domain is 0.71 Å, which is comparable to differences observed between structures of a given protein in different crystal forms. Hence, the differences between the conformations of the synaptotagmins-7 and -1 C_2_B domains can be considered to be within experimental error.

Comparison of the crystal structure of the synaptotagmin-7 C_2_B domain described here with the NMR structure of the synaptotagmin-1 C_2_B domain [17] leads to analogous conclusions to those drawn when we used the X-ray structure of the latter for the comparison. A backbone superposition of the three structures (Figure 4D) did suggest that slight differences between the conformations of Ca^2+^-binding loops 1 and 3 in the synaptotagmin-7 C_2_B domain (blue) and the synaptotagmin-1 C_2_B domain structures obtained by X-ray (orange) or NMR (yellow) might be meaningful, since the conformations of these loops in the two synaptotagmin-1 C_2_B domain structures are clearly more similar to each other than to their conformations in the synaptotagmin-7 C_2_B domain. However, loops 1 and 3 are known to have some degree of flexibility in C_2_ domains due to their exposed nature, and they are involved in extensive crystal contacts for the synaptotagmin-7 C_2_B domain (Figure 4E). Hence, the slight differences in the conformations of these loops are likely to arise from the crystal contacts, and it seems highly unlikely that they underlie the dramatic functional differences observed between synaptotagmins-1 and -7.

### The synaptotagmin-7 C_2_B domain binds three Ca^2+^ ions

The presence of Ca^2+^ in the conditions used to crystallize the synaptotagmin-7 C_2_B domain allowed us to determine its Ca^2+^-binding mode and to compare it with those determined previously for synaptotagmins-1 and -3. We observed three Ca^2+^ ions bound at the cup shape formed by the top loops of the synaptotagmin-7 C_2_B domain (loops 1–3, Figure 4A). We also observed a fourth Ca^2+^ ion bound outside of this region, but this site is not common in C_2_ domains [33] and the Ca^2+^ ion is coordinated by only three protein ligands, suggesting that occupation of this site is due to the high Ca^2+^ concentration present in the crystallization conditions (20 mM) and is not physiologically relevant.

**Figure 5:**
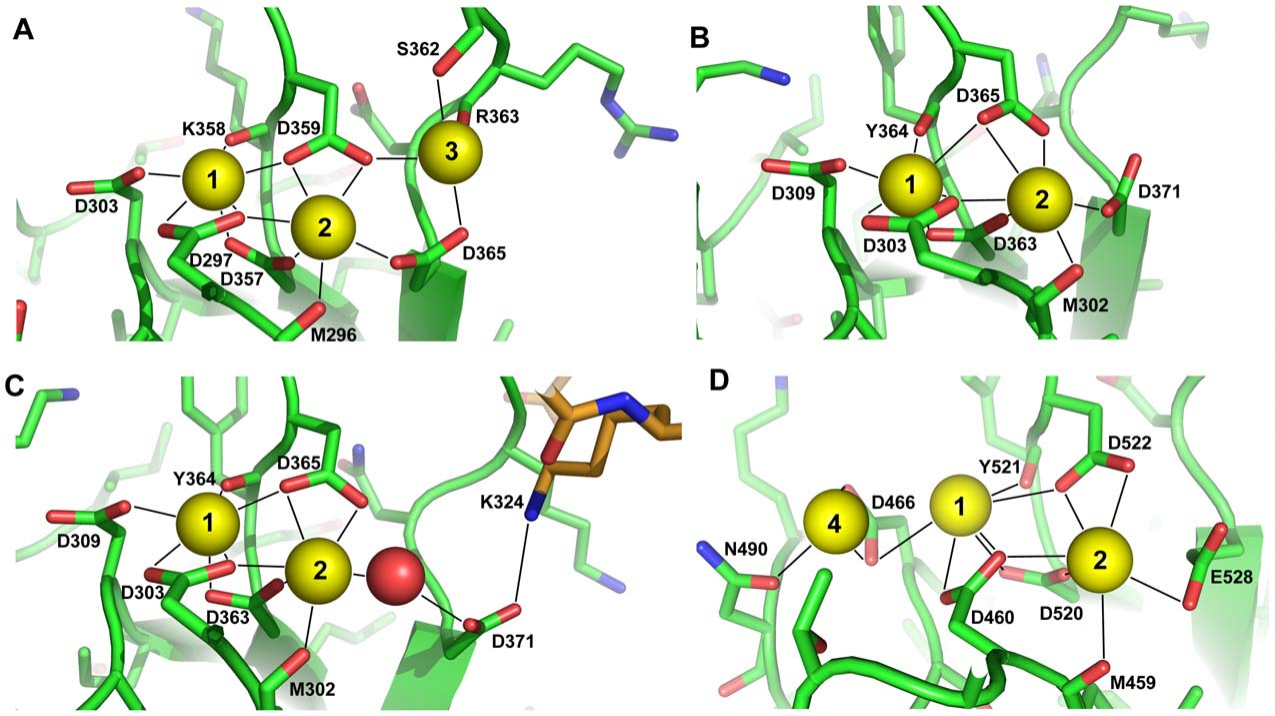
Comparison of the Ca^2+^-binding modes of the C_2_B domains of synaptotagmins-1, -3 and -7. (A) Ribbon diagram showing the Ca^2+^-binding mode of the synaptotagmin-7 C_2_B domain, with the Ca^2+^ ligands shown as color-coded stick models (green, carbon; blue, nitrogen; red, oxygen) and the bound Ca^2+^ ions shown as yellow spheres. (B) Ribbon diagram showing the Ca^2+^-binding mode of the synaptotagmin-1 C_2_B domain determined by NMR spectroscopy [17] (PDB accession code 1K5W). (C) Ribbon diagram showing the Ca^2+^-binding mode of the synaptotagmin-1 C_2_B domain determined by X-ray crystallography [43] (PDB accession code 1UOW). (D) Ribbon diagram showing the Ca^2+^-binding mode of the synaptotagmin-3 C_2_B domain in the X-ray structure of a synaptotagmin-3 C_2_AB fragment bound to Ca^2+^ [47] (PDB accession code 3HN8). The Ca^2+^-binding sites in the different structures are numbered from 1 to 4 according to the nomenclature of ref. [33], with the same numbers used for homologous sites. The color coding in (B–D) is the same as in panel (A), except that in (C) a small portion of a stick model of a second C_2_B domain molecule within the crystal is shown with carbon atoms colored in orange. The side chain of K324 in this region of the second C_2_B domain molecule makes a salt bridge with D371 of the central molecule as a result of crystal packing. A water molecule that coordinates Ca^2+^ ion 2 and serves as a bridge with the D371 side chain is shown as a red sphere. Note also that the serine side chain in loop 3 of the synaptotagmin-7 C_2_B domain (S362 panel A) is not present in the synaptotagmins-1 and -3 C_2_B domains (see Figure 2A) and, as a result, Ca^2+^ does not bind to site 3 (B,C).

The three Ca^2+^ ions bound at the cavity formed by the top loops are coordinated by five aspartate and one serine side chains, as well as by three backbone carbonyl groups (Figure 5A; the Ca^2+^-binding sites are numbered 1–3 as is common for C_2_ domains [33]). This binding mode is analogous to that observed for the synaptotagmin-1 C_2_A domain [15], [16]. In contrast, the C_2_B domain of synaptotagmin-1 was found to bind only two Ca^2+^ ions [17], [43] (Figures 5B–C) due to the absence of the serine side chain in loop 3 that helps to coordinate the third Ca^2+^ ion in the synaptotagmin-1 C_2_A domain (S235) and the synaptotagmin-7 C_2_B domain (S362). Note that this serine residue of loop 3 is not generally conserved in synaptotagmin C_2_B domains (Figure 2A) and hence binding of the third Ca^2+^ ion is an unusual feature of the synaptotagmin-7 C_2_B domain compared to other isoforms. Correspondingly, no Ca^2+^ binding to this site of the C_2_B domain was observed in the recently determined crystal structure of a synaptotagmin-3 C_2_AB fragment [47] (Figure 5D). A third bound Ca^2+^ ion was found in this region (labeled 4 in Figure 5D), but this ion is coordinated by only three protein ligands.

### Implications for the Ca^2+^-binding mode of the synaptotagmin-1 C_2_B domain

Despite the difference involving the third Ca^2+^-binding site, the ligands involved in coordinating the Ca^2+^ ions in sites 1 and 2 of the synaptotagmin-7 C_2_B domain (Figure 5A; coordinating waters not shown) are analogous to those determined to be involved in Ca^2+^-binding to the synaptotagmin-1 C_2_B domain in solution by NMR spectroscopy [17] (Figure 5B). It is worth noting that a crystal structure of the synaptotagmin-1 C_2_B domain [43] concluded that one of the aspartate side chains (D371) does not coordinate directly the Ca^2+^ ion in site 2, since a water molecule was inserted between this Ca^2+^ ion and D371 (Figure 5C). However, despite the high resolution of this crystal structure (1.04 Å), there was clear evidence for a potential distortion of the Ca^2+^-binding site by crystal contacts, since D371 interacts with a lysine (K324) from a neighboring molecule in this structure (Figure 5C). Hence, it seems most likely that the correct binding mode of the synaptotagmin-1 C_2_B domain is that determined in solution by NMR spectroscopy [17], which is now further supported by the crystal structure of the synaptotagmin-7 C_2_B domain presented here (see discussion).

## Discussion

Ca^2+^-dependent exocytosis is known to depend on diverse Ca^2+^ sensors that most likely emerged to meet distinct regulatory requirements for different types of cells and secretory processes. It also seems clear that at least some of the Ca^2+^ sensors belong to the synaptotagmin family [4]. However, the basic principles underlying functional differentiation among different synaptotagmin isoforms are not well understood, which is particularly well emphasized by the findings that synaptotagmins-1 and -7 both function as Ca^2+^ sensors in chromaffin cells [12], whereas neurotransmitter release in central nervous system synapses is triggered by synaptotagmin-1 but not by synaptotagmin-7 [5], [40]. To shed light into these principles, in this work we combined structural studies by X-ray crystallography with a rescue approach using a Syt1/7 chimera that cannot rescue neurotransmitter release in synaptotagmin-1 neurons, and attempted to endow the chimera with rescue activity with mutations that make the synaptotagmin-7 C_2_B domain more similar to the synaptotagmin-1 C_2_B domain. Although we were unable to obtain rescue activity, our results yield the crystal structure of the synaptotagmin-7 C_2_B domain and a number of insights into structure-function relationships in synaptotagmins.

First, our data confirm and extend previous results that revealed the central importance of the C_2_B domain for synaptotagmin-1 function. In previous studies, disrupting Ca^2+^ binding to synaptotagmin-1 C_2_B domain was found to abolish neurotransmitter release [21], [22], whereas disrupting Ca^2+^ binding to the C_2_A domain had milder effects [23], [24]. Moreover, mutating two arginines at the bottom of the C_2_B domain (R398 and R399) also abolished synaptotagmin-1 function, showing that not only the top, Ca^2+^-dependent face, but also the bottom, Ca^2+^-independent face of the C_2_B domain plays a key role in release [27]. Notably, one of these two arginines (R399) is a glutamine in synaptotagmin-7 (Q393) (see Figure 2), which suggested a simple explanation for the lack of rescue of neurotransmitter release by the Syt1/7 chimera, but replacing this glutamine with arginine (Q393R mutation) did not confer rescue to the chimera. Indeed, most of the mutant chimeras that we designed (mutants 1–9; Figure 2C) contain all the Ca^2+^ ligands at the top loops and the two arginines at the bottom face of the C_2_B domain, and yet they are unable to rescue neurotransmitter release in *synaptotagmin-1* KO neurons. It is important to note that the inability of at least some of the Syt1/7 chimeras to rescue fast release might arise because of improper protein targeting and/or folding. However, our immunocytochemistry data show that the chimeras were targeted to synapses, and previous results with different synaptotagmin-1/7 chimeras showed that including the N-terminal sequence of synaptotagmin-1 that contains an N-glycosylation site is sufficient for targeting to vesicles [48]. In fact, synaptotagmin-1 appears to be functional even if it is targeted to the plasma membrane [46]. Moreover, it seems highly unlikely that the point mutations we introduced in the Syt1/7 chimera, or replacement of the linker region, might cause folding problems because all these mutated residues are surface exposed. Even the two loop replacements involved mostly exposed residues and conservative substitutions of buried side chains that are unlikely to severely destabilize the domain. Hence, although we cannot completely rule out folding or targeting problems for some of the chimeras, our data do suggest that the Ca^2+^-binding sites at the top face and the arginines at the bottom face of the C_2_B domain are necessary but not sufficient for synaptotagmin-1 like function.

A potential explanation for the inability of synaptotagmin-7 [8] or the Syt1/7 chimera (Fig. 1) to rescue synaptic exocytosis was the absence of helix HB in the C_2_B domain, which was recently proposed to be important for synaptotagmin-1 function [46]. However, our data (Fig. 3) clearly show that this helix has an inhibitory role in synaptotagmin-1. It is also noteworthy that synaptotagmins-1 and -7 are both able to bind Ca^2+^, phospholipids and SNAREs [14], [40], [44], [49], which are the primary biochemical activities generally believed to underlie synaptotagmin function. It might be argued that, since the polybasic region of the C_2_B domain seems to be involved in SNARE binding [31], [50] and the synaptotagmin-7 C_2_B domain contains a Lys to Glu substitution in this region (E318), perhaps this substitution leads to weaker SNARE binding. However, no substantial differences between synaptotagmins-1 and -7 in SNARE binding were observed in a previous comparative study [44], and replacing Glu318 with Lys did not enable rescue activity on the Syt1/7 chimera (mutants 6–9; see Figures 1, 2C). Hence, these observations suggest that Ca^2+^, phospholipid and SNARE binding might not be sufficient for synaptotagmin-1 function. Clearly, further research will be necessary to test this possibility. For instance, synaptotagmin-1 appears to bind to the SNAREs in diverse modes, including an interaction with the syntaxin-1/SNAP-25 heterodimer that promotes vesicle docking [42], [51], but the exact nature of these interactions and the corresponding differences between synaptotagmin isoforms remain unclear.

Although we were unable to achieve rescue of fast neurotransmitter release in synaptotagmin-1 KO neurons with the Syt1/7 chimeras, our experiments with multiple mutants underscore how difficult it is to confer a specific activity to a protein isoform even when the starting point is a closely related isoform. Although this conclusion might seem trivial, it has not been sufficiently appreciated in the past to the best of our knowledge. Note that no rescue was observed even when we restored 30 residues of the chimera C_2_B domain back to those present in synaptotagmin-1 (mutant #9), since the resulting C_2_B domain is highly similar to the synaptotagmin-1 C_2_B domain and contains all the residues that have been identified so far to be critical for synaptotamin-1 function. Moreover, the crystal structure of the synaptotagmin-7 C_2_B domain described here shows that its backbone structure and even the conformations of many side chains are very similar to those of the synaptotagmin-1 C_2_B domain, suggesting that their functional differences do not arise from distinct structural properties. In contrast, the synaptotagmin-4 C_2_B domain was indeed found to have marked conformational differences with respect to the synaptotagmin-1 C_2_B domain that explained the inability of the former to bind Ca^2+^ [39]. Note that the synaptotagmin-4 C_2_B domain has a full complement of Ca^2+^ ligands (Figure 2), and that the C_2_B domains of synaptotagmins-1 and -4 have a similar degree of sequence identity (45%) to that between the C_2_B domains of synaptotagmins-1 and -7 (48%). All of these observations show that, although sequence analyses are sometimes very useful to predict biochemical and functional properties of proteins, such properties are often very difficult to predict and rationalize from the sequences alone. It is tempting to speculate that subtle sequence and/or structural distinctions among isoforms might be key to dictate dramatic functional differences.

The similarity between the crystal structure of the synaptotagmin-7 C_2_B domain described here and that of the synaptotagmin-1 C_2_B domain (Figure 4) illustrates how the structures of homologous proteins can be conserved exquisitely well, even in the side chain conformations. This high similarity shows that there is no obvious structural reason for the inability of synaptotagmin-7 to functionally replace synaptotagmin-1. We did observe a distinction between their C_2_B domains with respect to Ca^2+^ binding, since the synaptotagmin-7 C_2_B domain binds one more Ca^2+^ ion than the synaptotagmin-1 C_2_B domain due to the presence of a serine in loop 3 (Figure 5). The observation of this third Ca^2+^ binding site shows that there can be variability in the number of Ca^2+^-binding sites in Ca^2+^-dependent synaptotagmins. In principle, this distinction could provide a potential explanation for the lack of rescue of fast neurotransmitter release by the Syt1/7 chimera. However, it seems more likely that binding of the third Ca^2+^ ion might lead to a gain of function rather than to a complete loss of function.

Importantly, the ligands observed for the first two Ca^2+^-binding sites of the synaptotagmin-7 C_2_B domain are analogous to those originally identified by NMR spectroscopy as the Ca^2+^-ligands for the synaptotagmin-1 C_2_B domain [17] (Figure 5B), supporting the notion that this binding mode is indeed correct. While a high-resolution (1.04 Å) crystal structure of the synaptotagmin-1 C_2_B domain had suggested that one of the ligands (D371) does not directly coordinate Ca^2+^ [43], this side chain was involved in a crystal contact (Figure 5C). Moreover, this conclusion seemed to rely in part on the assumption that the NMR methodology used to determine the original structure of the synaptotagmin-1 C_2_B domain cannot determine Ca^2+^-binding modes accurately [43]. However, the 1.04 Å crystal structure itself provided strong evidence for the overall high quality of the NMR structure, since both structures were highly similar (1.0 Å r.m.s. deviation for 996 common atoms) and only differed slightly in the conformations of flexible loop regions, including the loop that contains D371 and is involved in crystal contacts. Furthermore, the location of the D371 side chain in the NMR structure was dictated not only by the mere assumption that this side chain coordinates Ca^2+^, as implied in ref. [43], but also by multiple restraints derived from nuclear Overhauser enhancements [17]. Note also that the Ca^2+^-binding modes of both synaptotagmin-1 C_2_ domains determined with our NMR methodology [15]–[17] have been corroborated by multiple crystal structures of homologous C_2_ domains (e.g. refs. [47], [52], [53] and Figures 5A,D), and that the side chain corresponding to D371 coordinates the Ca^2+^ ion in site 2 in all determined structures of C_2_ domains that bind Ca^2+^ at this site, with the exception of the 1.04 Å crystal structure of the synaptotagmin-1 C_2_B domain. From all these observations, it seems almost certain that the Ca^2+^-binding mode in the 1.04 Å crystal structure was distorted via lattice contact, and that D371 acts indeed as a direct Ca^2+^ ligand in synaptotagmin-1 in solution and in vivo.

## Methods

### Lentivirus constructs and production

A modified lentiviral vector [27], [54] was used, in which a human s*ynapsin-1* promoter and a *ubiquitin C* promoter drive the expression of synaptotagmin variants and the reporter, enhanced green fluorescent protein (EGFP), respectively. The same vector without synaptotagmin served as a control construct. The cDNA of wildtype mouse synaptotagmin-1 was cloned into the lentiviral vector and was used to generate synaptotagmin-1 delta HB mutant by standard recombinant DNA techniques. The cDNAs of wildtype rat synaptotagmin-1 and synaptotagmin-7 were used to generate chimeric synapotagmin-1/7 variants, which were subsequently cloned into the lentiviral vector. Lentiviruses were produced by co-transfecting HEK 293T cells with the lentiviral vector and two helper vectors, pVSVg and pCMV-delta R8.9 vector [54]. Viral supernatants were collected 48–72 hours after transfection and virus particles were concentrated using a centrifugal filter device (Amicon Ultra-15, Millipore).

### Mice, neuronal cultures, immunocytochemistry and Western blotting

*Synaptotagmin-1* knockout mice were obtained by interbreeding of mice heterozygous for the *synaptotagmin-1* mutation as described [55]. All procedures to maintain and use these mice were approved by the Institutional Animal Care and Use Committee for Baylor College of Medicine and Affiliates.

Primary neuronal cultures were prepared as described [56]. Briefly, hippocampal neurons were prepared from postnatal day 0 mice and plated at the density of 300 cm^−2^ on WT astrocyte microislands to obtain autaptic neurons for electrophysiology and immunocytochemistry experiments. Neurons were plated at the density of 10,000 cm^−2^ on confluent WT astrocytes for Western blot experiments.

Immunocytochemistry was performed as described [56] with modifications. Briefly, cultured neurons at day *in vitro* 10 were fixed with 4% (w/v) paraformaldehyde for 30 minutes at room temperature and the following primary antibodies (all from Synaptic Systems) were used: rabbit anti-GFP (1∶1000), guinea pig anti-vesicular glutamate transporter-1 (1∶1000), rabbit anti-synaptophyin-1 (1∶1000), and mouse anti-synaptotagmin-1 (clone 41.1, 1∶200). Images were acquired on a Leica TCS SP5 laser-scanning confocal microscope or a Zeiss 510 laser scanning confocal microscope. The settings for laser power and detector gain allowed the pixel intensities to remain within the dynamic range. Images were acquired as z-stacks with 8-bit and 1024×1024 pixel resolution and processed using software NIH ImageJ 1.40 g to create sum projections from the stacks.

Western blotting was performed as described [27] with mouse anti-synaptotagmin-1 (clone 41.1, 1∶12,000) and mouse anti-β-Tubulin III (clone 3B11, 1∶5,000, both antibodies from Synaptic Systems).

### Electrophysiology of cultured neurons

Whole-cell voltage clamp experiments were performed as described [57] on autaptic hippocampal glutamatergic neurons at room temperature (23–24°C). The extracellular solution contained (mM): NaCl, 140; KCl, 2.4; HEPES, 10; glucose, 10; CaCl_2_, 4 (for Figure 1A,B) or 2 (for Figures 1E,F and 3); MgCl_2_, 4; 300 mOsm; pH 7.4. The patch pipette solution contained (mM): KCl, 136; HEPES, 17.8; EGTA, 1; MgCl_2_, 0.6; ATP-Mg, 4; GTP-Na, 0.3; Phosphocreatine, 12; Phosphocreatine kinase, 50U ml^−1^; 300 mOsm; pH 7.4. Action potential-evoked EPSC, readily releasable pool size, vesicular release probability, short-term plasticity, and apparent Ca^2+^ sensitivity of release were measured as described [57]. Data were analyzed offline using AxoGraph X (AxoGraph Scientific). Statistic significances were tested using Student's *t*-test, one-way analysis of variance, or two-way analysis of variance.

### Crystallization of the synaptotagmin-7 C2B Domain

The vector to express the mouse synaptotagmin-7 C_2_B domain as a GST-fusion protein was described previously [58]. Bacterial expression of the synaptotagmin-7 C_2_B (residues 266–403), cleavage from the GST moiety and purification were performed as described previously for the synaptotagmin-1 C_2_B domain [59]. For crystallization, purified synaptotagmin-7 C_2_B domain was concentrated to 40 mg/ml in 25 mM NaAcetate buffer pH 6.2, 50 mM KCl, 20 mM CaCl2, and 1 mM TCEP. After concentration, a UV spectrum was recorded to determine protein concentration and ensure that the protein was not contaminated by DNA/RNA [59]. This protein solution was used for crystallization trials. Large multiple crystals were grown in 0.2M Li2SO4, 0.1M HEPES pH 6.5, and 25% (w/v) PEG 3350. These crystals were cryoprotected with 15% (v/v) ethylene glycol and flash-cooled in liquid nitrogen.

### Data Collection, Model Building, and Refinement

The crystallographic data were indexed and integrated using HKL3000 [60]. The structure was phased using the molecular replacement method with the synaptotagmin-1 C_2_B domain structure (PDB accession code 1TJM) as a search model. 136 of 138 residues were automatically built into the electron density and the sequence was assigned by the program ARP/warp [61], and the remaining N- and C-terminal residues were manually modeled into the electron density via the program COOT [62]. Refinement was performed using multiple rounds of PHENIX [63] alternated by model re-building in COOT. The final model for the synaptotagmin-7 C_2_B domain contains residues 266––403, 4 Ca^2+^ ions and 183 waters (final R_work_ = 16.0; R_free_ = 18.1; overall B-factor = 16.0). For data collection and refinement statistics, see **Table 1**. In the final model, 95.9% of the residues are in the favored regions of the Ramachandran map, and 4.1% of the residues in the additionally allowed regions. Coordinates and structure factors have been deposited in the Protein Data Bank with accession code 3N5A.

## Supporting Information

Figure S1 Delta HB mutant synaptotagmin-1 is expressed at similar levels as WT synaptotagmin-1 and properly targets to presynaptic terminals. synaptotagmin-1 KO neurons were infected with lentiviruses expressing EGFP alone or Syt1 and EGFP together. (A) Exemplary Western blot showing similar expression levels of WT and delta HB Syt1. Neuron specific b-Tubulin III serves as loading controls. (B) Exemplary confocal images showing the presynaptic localization of WT and delta HB Syt1. Neurons were immunostained with antibodies against the N-terminal portion of the Syt1 C2A domain (magenta images), synaptophysin-1 (green images), and EGFP (grey images). The merged images show the presence of WT and delta HB Syt1 in the presynaptic termini identified by synaptophysin-1. Scale bars: 10 mm.
(1.84 MB PDF)

## References

[1] Jahn R, Scheller RH (2006). SNAREs—engines for membrane fusion.. Nat Rev Mol Cell Biol.

[2] Rizo J, Rosenmund C (2008). Synaptic vesicle fusion.. Nat Struct Mol Biol.

[3] Sudhof TC, Rothman JE (2009). Membrane fusion: grappling with SNARE and SM proteins.. Science.

[4] Sudhof TC (2002). Synaptotagmins: why so many?. J Biol Chem.

[5] Fernandez-Chacon R, Konigstorfer A, Gerber SH, Garcia J, Matos MF (2001). Synaptotagmin I functions as a calcium regulator of release probability.. Nature.

[6] Rhee JS, Li LY, Shin OH, Rah JC, Rizo J (2005). Augmenting neurotransmitter release by enhancing the apparent Ca2+ affinity of synaptotagmin 1.. Proc Natl Acad Sci U S A.

[7] Pang ZP, Sun J, Rizo J, Maximov A, Sudhof TC (2006). Genetic analysis of synaptotagmin 2 in spontaneous and Ca2+-triggered neurotransmitter release.. EMBO J.

[8] Xu J, Mashimo T, Sudhof TC (2007). Synaptotagmin-1, -2, and -9: Ca(2+) sensors for fast release that specify distinct presynaptic properties in subsets of neurons.. Neuron.

[9] Gustavsson N, Lao Y, Maximov A, Chuang JC, Kostromina E (2008). Impaired insulin secretion and glucose intolerance in synaptotagmin-7 null mutant mice.. Proc Natl Acad Sci U S A.

[10] Michaut M, De BG, Tomes CN, Yunes R, Fukuda M (2001). Synaptotagmin VI participates in the acrosome reaction of human spermatozoa.. Dev Biol.

[11] Lynch KL, Martin TF (2007). Synaptotagmins I and IX function redundantly in regulated exocytosis but not endocytosis in PC12 cells.. J Cell Sci.

[12] Schonn JS, Maximov A, Lao Y, Sudhof TC, Sorensen JB (2008). Synaptotagmin-1 and -7 are functionally overlapping Ca2+ sensors for exocytosis in adrenal chromaffin cells.. Proc Natl Acad Sci U S A.

[13] Sutton RB, Davletov BA, Berghuis AM, Sudhof TC, Sprang SR (1995). Structure of the first C2 domain of synaptotagmin I: a novel Ca2+/phospholipid-binding fold.. Cell.

[14] Shao X, Davletov BA, Sutton RB, Sudhof TC, Rizo J (1996). Bipartite Ca2+-binding motif in C2 domains of synaptotagmin and protein kinase C.. Science.

[15] Ubach J, Zhang X, Shao X, Sudhof TC, Rizo J (1998). Ca2+ binding to synaptotagmin: how many Ca2+ ions bind to the tip of a C2-domain?. EMBO J.

[16] Shao X, Fernandez I, Sudhof TC, Rizo J (1998). Solution structures of the Ca2+-free and Ca2+-bound C2A domain of synaptotagmin I: does Ca2+ induce a conformational change?. Biochemistry.

[17] Fernandez I, Arac D, Ubach J, Gerber SH, Shin O (2001). Three-dimensional structure of the synaptotagmin 1 c(2)b-domain. Synaptotagmin 1 as a phospholipid binding machine.. Neuron.

[18] Ubach J, Garcia J, Nittler MP, Sudhof TC, Rizo J (1999). Structure of the Janus-faced C2B domain of rabphilin.. Nat Cell Biol.

[19] Chapman ER, Davis AF (1998). Direct interaction of a Ca2+-binding loop of synaptotagmin with lipid bilayers.. J Biol Chem.

[20] Zhang X, Rizo J, Sudhof TC (1998). Mechanism of phospholipid binding by the C2A-domain of synaptotagmin I.. Biochemistry.

[21] Mackler JM, Drummond JA, Loewen CA, Robinson IM, Reist NE (2002). The C(2)B Ca(2+)-binding motif of synaptotagmin is required for synaptic transmission in vivo.. Nature.

[22] Nishiki T, Augustine GJ (2004). Dual roles of the C2B domain of synaptotagmin I in synchronizing Ca2+-dependent neurotransmitter release.. J Neurosci.

[23] Fernandez-Chacon R, Shin OH, Konigstorfer A, Matos MF, Meyer AC (2002). Structure/function analysis of Ca2+ binding to the C2A domain of synaptotagmin 1.. J Neurosci.

[24] Stevens CF, Sullivan JM (2003). The synaptotagmin C2A domain is part of the calcium sensor controlling fast synaptic transmission.. Neuron.

[25] Arac D, Chen X, Khant HA, Ubach J, Ludtke SJ (2006). Close membrane-membrane proximity induced by Ca(2+)-dependent multivalent binding of synaptotagmin-1 to phospholipids.. Nat Struct Mol Biol.

[26] Rizo J, Chen X, Arac D (2006). Unraveling the mechanisms of synaptotagmin and SNARE function in neurotransmitter release.. Trends Cell Biol.

[27] Xue M, Ma C, Craig TK, Rosenmund C, Rizo J (2008). The Janus-faced nature of the C(2)B domain is fundamental for synaptotagmin-1 function.. Nat Struct Mol Biol.

[28] Bhalla A, Chicka MC, Tucker WC, Chapman ER (2006). Ca(2+)-synaptotagmin directly regulates t-SNARE function during reconstituted membrane fusion.. Nat Struct Mol Biol.

[29] Tang J, Maximov A, Shin OH, Dai H, Rizo J (2006). A complexin/synaptotagmin 1 switch controls fast synaptic vesicle exocytosis.. Cell.

[30] Bowen ME, Weninger K, Ernst J, Chu S, Brunger AT (2005). Single-molecule studies of synaptotagmin and complexin binding to the SNARE complex.. Biophys J.

[31] Dai H, Shen N, Arac D, Rizo J (2007). A Quaternary SNARE-Synaptotagmin-Ca(2+)-Phospholipid Complex in Neurotransmitter Release.. J Mol Biol.

[32] Choi UB, Strop P, Vrljic M, Chu S, Brunger AT (2010). Single-molecule FRET-derived model of the synaptotagmin 1-SNARE fusion complex.. Nat Struct Mol Biol.

[33] Rizo J, Sudhof TC (1998). C2-domains, structure and function of a universal Ca2+-binding domain.. J Biol Chem.

[34] Chapman ER (2008). How Does Synaptotagmin Trigger Neurotransmitter Release?. Annu Rev Biochem.

[35] Corbalan-Garcia S, Gomez-Fernandez JC (2010). The C2 domains of classical and novel PKCs as versatile decoders of membrane signals.. Biofactors.

[36] Dulubova I, Lou X, Lu J, Huryeva I, Alam A (2005). A Munc13/RIM/Rab3 tripartite complex: from priming to plasticity?. EMBO J.

[37] Lu J, Machius M, Dulubova I, Dai H, Sudhof TC (2006). Structural Basis for a Munc13-1 Homodimer to Munc13-1/RIM Heterodimer Switch.. PLoS Biol.

[38] Garcia J, Gerber SH, Sugita S, Sudhof TC, Rizo J (2004). A conformational switch in the Piccolo C2A domain regulated by alternative splicing.. Nat Struct Mol Biol.

[39] Dai H, Shin OH, Machius M, Tomchick DR, Sudhof TC (2004). Structural basis for the evolutionary inactivation of Ca2+ binding to synaptotagmin 4.. Nat Struct Mol Biol.

[40] Maximov A, Lao Y, Li H, Chen X, Rizo J (2008). Genetic analysis of synaptotagmin-7 function in synaptic vesicle exocytosis.. Proc Natl Acad Sci U S A.

[41] Gerber SH, Rah JC, Min SW, Liu X, de WH (2008). Conformational switch of syntaxin-1 controls synaptic vesicle fusion.. Science.

[42] de WH, Walter AM, Milosevic I, Gulyas-Kovacs A, Riedel D (2009). Synaptotagmin-1 docks secretory vesicles to syntaxin-1/SNAP-25 acceptor complexes.. Cell.

[43] Cheng Y, Sequeira SM, Malinina L, Tereshko V, Sollner TH (2004). Crystallographic identification of Ca2+ and Sr2+ coordination sites in synaptotagmin I C2B domain.. Protein Sci.

[44] Rickman C, Craxton M, Osborne S, Davletov B (2004). Comparative analysis of tandem C2 domains from the mammalian synaptotagmin family.. Biochem J.

[45] Bhalla A, Chicka MC, Chapman ER (2008). Analysis of the Synaptotagmin Family during Reconstituted Membrane Fusion: UNCOVERING A CLASS OF INHIBITORY ISOFORMS.. J Biol Chem.

[46] Hui E, Johnson CP, Yao J, Dunning FM, Chapman ER (2009). Synaptotagmin-mediated bending of the target membrane is a critical step in Ca(2+)-regulated fusion.. Cell.

[47] Vrljic M, Strop P, Ernst JA, Sutton RB, Chu S (2010). Molecular mechanism of the synaptotagmin-SNARE interaction in Ca2+-triggered vesicle fusion.. Nat Struct Mol Biol.

[48] Han W, Rhee JS, Maximov A, Lao Y, Mashimo T (2004). N-glycosylation is essential for vesicular targeting of synaptotagmin 1.. Neuron.

[49] Hui E, Bai J, Wang P, Sugimori M, Llinas RR (2005). Three distinct kinetic groupings of the synaptotagmin family: candidate sensors for rapid and delayed exocytosis.. Proc Natl Acad Sci U S A.

[50] Rickman C, Archer DA, Meunier FA, Craxton M, Fukuda M (2004). Synaptotagmin interaction with the syntaxin/SNAP-25 dimer is mediated by an evolutionarily conserved motif and is sensitive to inositol hexakisphosphate.. J Biol Chem.

[51] Lee HK, Yang Y, Su Z, Hyeon C, Lee TS (2010). Dynamic Ca2+-dependent stimulation of vesicle fusion by membrane-anchored synaptotagmin 1.. Science.

[52] Sutton RB, Sprang SR (1998). Structure of the protein kinase Cbeta phospholipid-binding C2 domain complexed with Ca2+.. Structure.

[53] Verdaguer N, Corbalan-Garcia S, Ochoa WF, Fita I, Gomez-Fernandez JC (1999). Ca(2+) bridges the C2 membrane-binding domain of protein kinase Calpha directly to phosphatidylserine.. EMBO J.

[54] Lois C, Hong EJ, Pease S, Brown EJ, Baltimore D (2002). Germline transmission and tissue-specific expression of transgenes delivered by lentiviral vectors.. Science.

[55] Geppert M, Goda Y, Hammer RE, Li C, Rosahl TW (1994). Synaptotagmin I: a major Ca2+ sensor for transmitter release at a central synapse.. Cell.

[56] Xue M, Reim K, Chen X, Chao HT, Deng H (2007). Distinct domains of complexin I differentially regulate neurotransmitter release.. Nat Struct Mol Biol.

[57] Xue M, Stradomska A, Chen H, Brose N, Zhang W (2008). Complexins facilitate neurotransmitter release at excitatory and inhibitory synapses in mammalian central nervous system.. Proc Natl Acad Sci U S A.

[58] Sugita S, Han W, Butz S, Liu X, Fernandez-Chacon R (2001). Synaptotagmin VII as a plasma membrane Ca(2+) sensor in exocytosis.. Neuron.

[59] Ubach J, Lao Y, Fernandez I, Arac D, Sudhof TC (2001). The C2B domain of synaptotagmin I is a Ca2+-binding module.. Biochemistry.

[60] Otwinowski Z, Minor W (1997). Processing of X-ray diffraction data collected in oscillation mode.. Macromolecular Crystallography.

[61] Potterton E, Briggs P, Turkenburg M, Dodson E (2003). A graphical user interface to the CCP4 program suite.. Acta Crystallogr D Biol Crystallogr.

[62] Emsley P, Cowtan K (2004). Coot: model-building tools for molecular graphics.. Acta Crystallogr D Biol Crystallogr.

[63] Adams PD, Afonine PV, Bunkoczi G, Chen VB, Davis IW (2010). PHENIX: a comprehensive Python-based system for macromolecular structure solution.. Acta Crystallogr D Biol Crystallogr.

