# Supplementary figures and images for: Structural and Mutational Analysis of Functional Differentiation between Synaptotagmins-1 and -7
Source: PLoS One. 2010 Sep 2;5(9):e12544. doi: 10.1371/journal.pone.0012544 (PMC2932738; doi:10.1371/journal.pone.0012544)

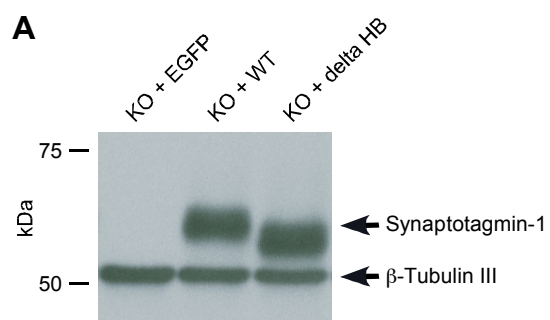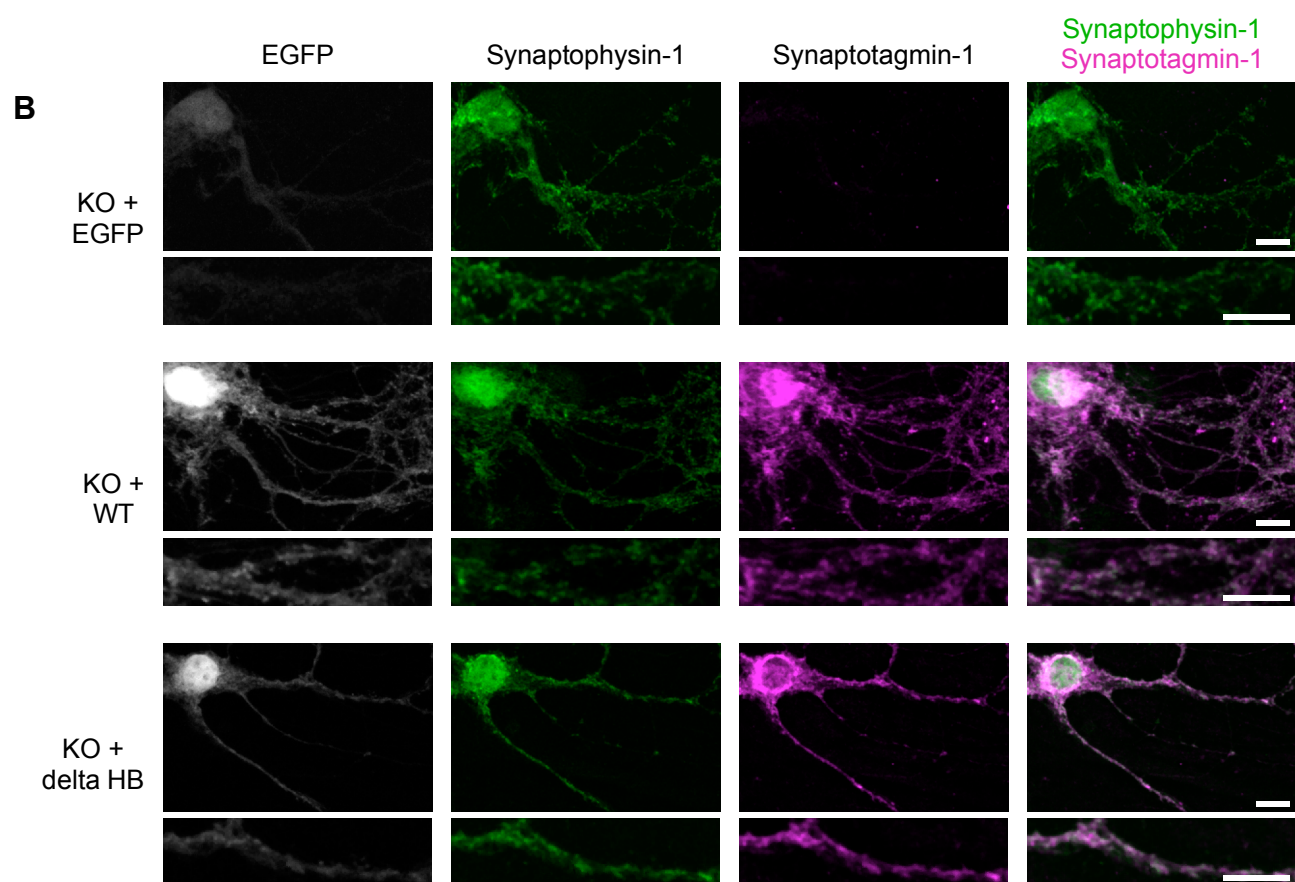

Supplement: Figure S1 — Delta HB mutant synaptotagmin-1 is expressed at similar levels as WT synaptotagmin-1 and properly targets to presynaptic terminals. synaptotagmin-1 KO neurons were infected with lentiviruses expressing EGFP alone or Syt1 and EGFP together. (A) Exemplary Western blot showing similar expression levels of WT and delta HB Syt1. Neuron specific b-Tubulin III serves as loading controls. (B) Exemplary confocal images showing the presynaptic localization of WT and delta HB Syt1. Neurons were immunostained with antibodies against the N-terminal portion of the Syt1 C2A domain (magenta images), synaptophysin-1 (green images), and EGFP (grey images). The merged images show the presence of WT and delta HB Syt1 in the presynaptic termini identified by synaptophysin-1. Scale bars: 10 mm. (1.84 MB PDF) [file pone.0012544.s001.pdf]
